# Lifestyle habits and use of general medical services among individuals with obsessive-compulsive disorder: a qualitative study

**DOI:** 10.1186/s12888-026-07965-7

**Published:** 2026-03-16

**Authors:** Sofia Asplund, Anna Holmberg, Klara Olofsdotter Lauri, Dante Lenninger, Ekaterina Ivanova, Matthias Lidin, Christian Rück, Lina Martinsson, David Mataix-Cols, Lorena Fernández de la Cruz

**Affiliations:** 1 Centre for Psychiatry Research, Department of Clinical Neuroscience, Karolinska Institutet, Stockholm, Sweden; 2 Stockholm Health Care Services, Region Stockholm, Stockholm, Sweden; 3 Division of Psychology, Department of Clinical Neuroscience, Karolinska Institutet, Stockholm, Sweden; 4 Department of Medicine, Karolinska Institutet, Stockholm, Sweden; 5 Department of Cardiology, Heart, Vascular and Neuro Theme, Karolinska University Hospital, Stockholm, Sweden; 6 Department of Clinical Sciences, Lund University, Lund, Sweden

**Keywords:** Obsessive-compulsive disorder, Lifestyle habits, Barriers, Lifestyle change, Healthcare services, Thematic analysis.

## Abstract

**Objectives:**

Although obsessive-compulsive disorder (OCD) is strongly associated with a range of modifiable somatic health problems, little is known about how the disorder influences the adoption and maintenance of healthy lifestyle habits, as well as how it shapes patients' experiences of seeking and receiving general healthcare.

**Methods:**

Sixteen individuals with OCD and cardiometabolic risk who participated in the piloting of a lifestyle intervention completed a semi-structured interview about the impact of OCD on their lifestyle habits and their experiences with general medical services. The interviews were analysed using reflexive thematic analysis.

**Results:**

The analysis generated three main themes and one overarching theme. The main themes were: (1) Living with multiple barriers to engage in healthy behaviours; (2) Changes in lifestyle habits – challenging but possible; and (3) OCD is a roadblock in general medical care. The overarching theme was: (4) It is not just OCD. The themes reflected that the participants experienced both disorder-specific and general barriers when trying to implement healthy lifestyle behaviours. Changing lifestyle habits was regarded as difficult, but facilitators of change were also identified. Participants reported that OCD affected seeking and receiving healthcare for their somatic problems. OCD was generally viewed as only one of many elements that affected health and lifestyle.

**Conclusions:**

Our results indicate the need for tailored support for this at-risk group to change and maintain healthy lifestyles, as well as a need of increasing knowledge of OCD among general medical care practitioners.

**Trial registration:**

Not applicable.

**Clinical trial number:**

Not applicable.

**Supplementary Information:**

The online version contains supplementary material available at 10.1186/s12888-026-07965-7.

## Background

Obsessive-compulsive disorder (OCD) is a chronic mental disorder with a prevalence of 1–2% in the general population [1]. OCD causes significant impact on quality of life and psychosocial functioning [2, 3], and is associated with long-term socio-economic adversity [4, 5]. The disorder has also been associated with a span of somatic conditions and increased risk of mortality due to both unnatural (e.g., suicide) and natural causes [6, 7].

Somatic conditions are here defined as communicable or non-communicable non-psychiatric bodily health conditions. Previous literature has described a robust association between OCD and non-communicable somatic conditions such as cardiometabolic and cerebrovascular disorders, including cardiovascular diseases, obesity, type 2 diabetes, stroke, and other circulatory system diseases [8–10]. In turn, most non-communicable somatic conditions have been associated with the presence of modifiable pernicious lifestyle habits. These lifestyle factors mostly include physical inactivity, unhealthy diets, use of tobacco, and harmful alcohol consumption [11–14]. The implementation of healthier lifestyle habits plays a significant role in the prevention of these conditions [15], also in individuals with serious mental illness [16].

Despite the scarcity of research, preliminary evidence indicates that individuals with OCD, as a group, tend to have unhealthy lifestyle habits. Compared with general population samples, people with OCD are more likely to report low levels of physical activity and unhealthy dietary habits, smoke more, sleep worse, and have a risk-level alcohol consumption [17–21]. In an international survey including about 500 participants reporting a diagnosis of OCD, 90% described to have considered changing their lifestyle habits and adopting healthier ones, with only about half of those succeeding in making lifestyle changes [21]. The precise reasons why individuals with OCD may have poorer lifestyles are not well understood.

There is compelling evidence indicating that individuals with mental disorders are less likely to obtain standard level of healthcare for several somatic problems [22]. However, in the case of OCD, knowledge on this topic is extremely scarce. Aguglia et al. [18] hypothesized that individuals with OCD might avoid seeking medical care because of specific obsessions (e.g., contamination fears), which would prevent diagnosing medical problems and the timely receipt of treatments. However, this is yet to be confirmed. Additionally, no previous research has investigated the experiences of people with OCD when seeking general medical care and being treated for somatic problems.

In this qualitative investigation, we interviewed 16 participants who had previously participated in a study testing the feasibility of a lifestyle intervention for individuals with OCD and cardiometabolic risk factors [23] to gather information on how OCD affected their lifestyle habits and their experiences of seeking and receiving general medical care.

## Methods

The study was approved by the Swedish Ethical Review Authority (reference numbers 2022-00375-01 and 2024-00708-02). All participants provided written informed consent to take part in the study. The study has been carried out considering the recommendations by Levitt et al. [24] for promoting methodological integrity in qualitative research and the criteria by Tracy [25] for best qualitative research practice. The consolidated criteria for reporting qualitative research (COREQ) have been used to clarify and report methodological choices [26] (see COREQ checklist in the Supplementary material).

### The LIFT feasibility trial

The current study is part of an open feasibility trial of a lifestyle intervention for individuals with OCD conducted in Stockholm (Sweden), described in detail elsewhere [23]. In brief, the intervention was delivered to four groups of 6–7 participants, resulting in the final inclusion of 25 adults with both OCD and increased risk of cardiometabolic risk factors (operationalized as having at least 3 out of a list of 11 predefined risk factors including, for example, physical inactivity, unhealthy diet, being a smoker, being overweight or obese or having hypertension). The trial aimed at examining the feasibility and acceptability, preliminary efficacy, and participants’ experiences of participation in a lifestyle intervention tailored to individuals with OCD, called LIFT (Swedish acronym for *Livsstilsintervention för tvångssyndrom* [Lifestyle intervention for OCD]). LIFT had a 3-month duration and included one individual session to set goals, six educational group sessions focusing on different lifestyle habits, and 12 group exercise sessions. Outcome measures included lifestyle habits, physiological parameters, and mental health symptoms and quality of life collected at baseline, post-intervention, and at the 3-month follow-up.

### Participants

Of the 25 participants included in the LIFT feasibility trial, 21 provided data at all time points and were subsequently invited via phone to take part in the qualitative interviews. Of these, 18 agreed to be interviewed, although two people were finally not reachable. The final sample included 16 individuals out of the initial 25 (64.0%). This number of interviews was deemed sufficient to respond to the research questions from an experiential perspective, answering the need for richness of information on the subject matter [27], rather than focusing on saturation [28]. See Table 1 for details on the characteristics of the participants.

**Table 1:** Characteristics of the sample (*N* = 16)

Variables	*n* (%)
**Demographic variables**	
Gender (women)	13 (81.2%)
Age (years), M (*SD*)	39.7 (11.2)
Age, min-max	23–60
**Occupational status**	
Disability pension/sick leave	5 (31.2%)
Vocational rehabilitation	2 (12.5%)
Student	3 (18.4%)
Full-time/part-time employment	6 (37.5%)
**OCD symptom severity** ^**1**^	
Y-BOCS, M (*SD)*	24.1 (5.7)
Mild	3 (18.7%)
Moderate	10 (62.5%)
Severe	3 (18.7%)
**C** **omorbidities** ^**1**^	
Psychiatric disorders	10 (62.5%)
Neurodevelopmental disorders^2^	6 (37.5%)
Anxiety-related disorders^3^	3 (18.7%)
Depression	2 (12.5%)
Somatic disorders	6 (37.5%)
Hypertension	4 (25.0%)
Type 2 diabetes	1 (6.2%)
Other somatic disorders	4 (25.0%)

Abbreviations: OCD, obsessive-compulsive disorder; Y-BOCS, Yale-Brown Obsessive-Compulsive Scale

^1^ As assessed in the LIFT feasibility trial baseline assessment. See Holmberg et al. [23] for further detail

^2^ Including autism spectrum disorder, attention-deficit/hyperactivity disorder, and/or Tourette syndrome

^3^ Including social anxiety disorder, posttraumatic stress disorder, and/or health anxiety disorder

### Qualitative interviews

The semi-structured, qualitative interviews included two parts. The first set of questions aimed at investigating the participants’ experiences of taking part of the LIFT intervention and gathered feedback that would allow to improve both the intervention and the study procedures in a subsequent larger randomized controlled trial. Results of this part of the qualitative interview are reported in the original publication [23]. The second part of the interview included seven questions, which are the focus of the current study. This second set of questions (available in the Supplementary material) aimed at exploring the lived experiences of individuals with OCD with regards to lifestyle and health-related behaviours.

The questions, and open follow-up questions, touched upon the reasons why they participated in the lifestyle intervention, whether and how OCD and psychiatric comorbidities impacted lifestyle habits and health-related behaviours. They also focused on the ability to make changes in relation to health behaviours, and whether the participants had experienced that their mental health had kept them from accessing healthcare and what had been the impact of this. The interview guide was developed by the first and second authors (SA and AH) and reviewed by the last author (LFC).

### Procedures

All interviews were conducted over the phone between February and May 2024. The interviews for this study were conducted right after the 3-month follow-up (*n* = 6) or up to 9 months after this timepoint (*n* = 10) by the first author (SA), a female clinical psychologist with considerable experience working with individuals with OCD, who had not had previous interaction with the participants. The average length of the interviews was 22.5 min, spanning between 12 and 50 min. All participants were alone at the point of the interview, except one who had their municipal housing support with them at the start of the call.

The interviews were audio-recorded, transcribed verbatim using a locally hosted Whisper model [29], and then revised for accuracy by authors SA and DL. The transcripts were not shown to the participants for corrections or additional comments. However, participants were told during the interviews that they were welcome to contact the team for additional comments. No participants contacted the interviewer for additional commentary, and no repeat interviews were conducted.

Reflexivity in the study was guided by Olmos-Vega et al. [30]. Throughout the interviews and analyses, the researchers worked to remain aware of own presumptions regarding the participants’ experiences through concurrent discussions on this topic. A reflexive journal was kept by the interviewer to collect her impressions throughout the data collection process and later inform the coding of the interviews, following the recommendations by Braun and Clarke [31] when utilizing reflexive thematic analysis. The interviewer made efforts to provide an open, non-judgemental, climate in the interviews and remain aware of her own status and preconceived notions or biases when analysing the data.

### Data analysis

Data were analysed using reflexive thematic analysis [31, 32]. The analysis was conducted between August and December 2024 using Microsoft Word and the NVivo 14 software.

The theoretical framework for the analysis was based on a critical-realist perspective [32], acknowledging that although reality exists independently, our understanding of reality is influenced by contextual factors. Thus, this study aims to report on the reality of the participants’ experiences as they were described, taking into account that social, cultural, and contextual factors may influence the way they view reality. The analysis was inductive, aiming to look at the descriptions of the participants’ own experiences without placing this within any wider theory during analysis. This approach was chosen since this is, to our knowledge, the first study to examine the experience of individuals with OCD with regards to lifestyle and health-related behaviours.

Since the data analysis was conducted by clinical psychologists who have extensive experience treating OCD, this will have influenced the output. This subjectivity was reflected upon, discussing how this could influence the analyses during the coding and mapping of themes, as the researchers were able to add their own framework to structure the data.

Following Braun and Clarke [31, 32], the analysis was conducted by moving back and forth through the steps for doing thematic analyses. The transcripts were firstly read and re-read by SA and AH for familiarization with the data. Coding was initiated by a joint meeting between SA, AH, and KOL, where the first two interviews were coded together by taking note of items bearing meaning within the texts and generating codes accordingly. The remaining transcripts were split evenly between SA and AH and coded separately. Codes were compared and discussed to reach consensus in the coding. The codes were then collated by SA. At this stage, the codes were reviewed together by SA, AH, and KOL to ensure depth and comprehensiveness of the codes.

Codes were then generated into meaningful themes by SA and AH, mapping themes and subthemes. These themes were continuously discussed with the research team, revising and refining the analysis to ensure that the themes were clearly grounded in the data, meaning contribution, and coherence of the themes in relation to the collected data [27]. Generating the themes was an iterative process, checking the themes repeatedly against the data, and stopping when no new themes or subthemes were generated. The themes were finally reviewed by AH against the codes and the entire dataset and then discussed with SA and KOL. After this, themes were adjusted to better match the data. Final adjustments were made by SA, AH, and LFC when writing the manuscript, and quotes were selected to illustrate the themes. This resulted in a final model accepted by the entire research team. Themes were then given names to represent the underlying idea of the generated theme. Finally, theme names and quotes were translated from Swedish into English for publication purposes.

## Results

**Fig. 1:**
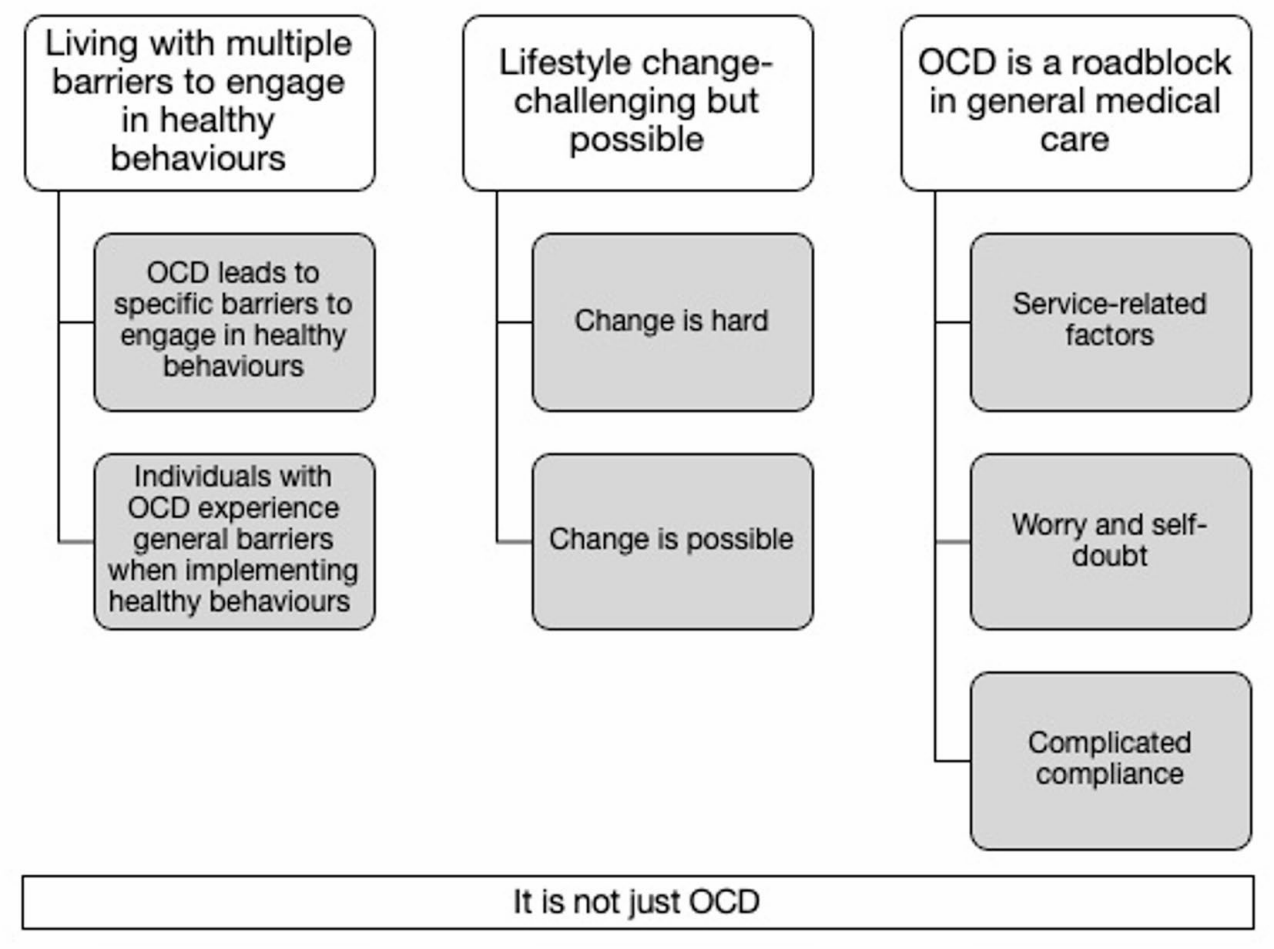
Generated themes, subthemes, and overarching theme

The reflexive thematic analysis generated three main themes and one overarching theme. The main themes were: (1) Living with multiple barriers to engage in healthy behaviours; (2) Lifestyle change – challenging but possible; and (3) OCD is a roadblock in general medical care. The overarching theme was: (4) It is not just OCD. During the reflexive process, the overarching theme was deemed most clearly influenced by clinical experience, as it echoed difficulties described by patients met in clinical practice. This subjectivity was seen as a helpful addition to the interpretation of the data. Themes, subthemes, and the overarching theme are presented below and in Fig. 1. Participants are presented as ID numbers 1 to 16.

### Living with multiple barriers to engage in healthy behaviours

The first main theme highlights the participants’ experiences of the barriers hindering them from engaging in healthy behaviours. The barriers were interpreted as being a direct or indirect result of the OCD, although more general barriers that could affect anyone, regardless of their mental health, were also present. The barriers also had an additive effect. For example, time-consuming compulsions in OCD can lead to being more susceptible to general barriers, such as not having time to exercise.

#### OCD leads to specific barriers to engage in healthy behaviours

All (*N* = 16) participants described that OCD had a direct practical impact on engaging in healthy behaviours. These included, for example, having to leave a certain environment or not being able to use knives to cut food, resulting in buying unhealthier ready-made alternatives.

*I used to eat very unhealthily. I preferred eating snacks rather than food*,* because it was easier for me than cooking a whole meal. It takes me twice as long to cook a meal. I need to cut things in a certain way. And I also don’t like when it gets dirty and messy*,* so I have to clean while cooking.* (ID 11, woman, age 20–30)

*When my OCD gets worse or really bad*,* I get thoughts about being dirty and I avoid touching things. So*,* I avoid doing exercise and touching things. Or it’s difficult for me to go to the laundry room because I can’t use it… so I try not using that many clothes so I don’t have to wash them*,* because I can’t go to the laundry room and wash them. If I would exercise*,* I would become sweaty and would have to wash my clothes.* (ID 6, woman, age 31–40)

All (*N* = 16) participants also described how OCD had an indirect effect on engaging in healthy habits, such as making things generally more tiring or more difficult, which in turn affected the probability of, for example, going to the gym.

*Maybe more like not having energy to exercise and so on*,* that you’re always tired. It is hard to relax while constantly thinking about these things* [obsessions]. (ID 10, man, age 20–30)

Further, OCD could lead to financial burden, which in turn may lead to a more general, economic barrier that affects the implementation of healthy behaviours.

*If someone coughed or sneezed close to my earphones on the subway*,* it would be very disturbing to me. It’s stupid. That was also a whole new thing. It gets very expensive to buy new earphones all the time.* (ID 14, woman, age 31–40)

*My obsessions have been about hurting people*,* and knives*,* and stuff like that. So I haven’t… I haven’t cooked much myself. I’ve eaten out. And it gets expensive.* (ID 7, man, age 31–40)

Another indirect effect of OCD on implementing healthy behaviours, reported by half of the participants (*n* = 8), was using unhealthy behaviours to reduce anxiety stemming from the OCD symptoms.

*I used to drink beer and eat pizza to relieve the anxiety I had because of my OCD.* (ID 5, woman, age 31–40)

*When it triggers a* [distressing] *thought*,* I get a craving for nicotine. I get so restless.* (ID 2, woman, age 41–50)

Finally, another eight participants noted that their lifestyle habits were affected given that OCD affected all aspects of their life.

*Exactly. I feel handicapped*,* like there’s a complete stop in… in my life.* (ID 6, woman, age 31–40)

*It has affected my whole life*,* besides exercise*,* like looking for new jobs*,* meeting a partner… It has affected me on all levels*,* not only related to exercise or well-being.* (ID 7, man, age 31–40)

#### Individuals with OCD also experience general barriers when implementing healthy behaviours

Thirteen of the participants gave examples of more general factors that influenced their health-related behaviours. The participants gave examples of practical barriers that any person might experience, such as pain, bad weather, or long traveling distances.

*Then it’s also winter or autumn; “Now you have to travel 45 minutes by bus at night when it’s freezing*,* and do heavy exercise and then go home”. That obstacle was so damn high…* (ID 4, man, age 41–50)

*I would have liked to have the energy to exercise but it’s difficult when I have my damn migraine.* (ID 16, woman, age 31–40)

Nine participants also noted general barriers, such as being too tired to exercise or not having enough time or money to engage in healthy behaviours. In turn, these general barriers could be exacerbated by the direct or indirect effects of OCD.

*I don’t have the energy*,* I’m tired*,* I might not have the energy to cook. Then you’ll end up eating some junk. *(ID 15, woman, age 31–40)

*It is expensive to have a gym card.* (ID 12, woman, age 51–60)

### Lifestyle change – challenging but possible

All participants had taken part in an intervention to change health-related behaviours and shared reflections on trying to change their lifestyle.

#### Change is hard

Twelve participants reflected on the difficulties to achieve behavioural changes when trying to modify lifestyle habits before, during, and after the intervention. In many ways, these interacted with the above-mentioned barriers to engage in healthy behaviours. Changing their lifestyle was generally described as difficult. Many recognized this pattern from previous attempts to initiate and maintain change.

*I really believed that*,* after this long lifestyle intervention*,* I would be able to make changes. But it seems to be terribly hard. I did not think that about myself. If it is due to OCD or something else I have*,* I don’t know.* (ID 1, woman, age 51–60)

Many barriers to change were more general, including age, time dedication, somatic problems or lack of motivation.

*Laziness*,* getting too comfortable*,* age… you become more set in your ways the older you get and it is more difficult to change*,* in my experience.* (ID 8, woman, age 41–50)

Six participants gave examples of their experiences regarding how OCD and mental health problems got in the way of making and maintaining long-term changes in health-related behaviours. Participants noted a connection to the above-mentioned OCD-specific barriers when engaging in healthy behaviours, but also a more general experience of OCD getting in the way of change.

*I’ve wanted to start exercising more and stuff like that. But it’s the same. It’s hard to go out because of the reasons I’ve… Yeah*, [the OCD] *is there again. It takes a lot of time. You get home from work and you’re supposed to go exercise*,* but you feel like you need to wash yourself first*,* and all of a sudden it’s been two hours.* (ID 10, man, age 20–30)

*There were tips and advice on how to eat healthier* [in the lifestyle intervention], *but my OCD got in the way*,* so I did not manage to change my habits.* (ID 8, woman, age 41–50)

#### Change is possible

All participants (*N* = 16) shared experiences regarding what had facilitated change for them, both within the context of the intervention and more generally. Individual adaptations were described as helpful.

*That’s why it was so nice that I was able to do this exercise at home. I just needed a mat*,* a resistance band*,* and we had some free weights at home. So I could do proper exercise at home. And that… that made it a bit easier. Because it is very difficult for me… I find it difficult to go to the gym.* (ID 15, woman, age 31–40)

*One thing I’ve noticed is that*,* since it’s hard for me to leave my home*,* regardless of whether it’s OCD or I have stuff to get done*,* I have walked at home. The times when I’ve thought I can’t manage to go out*,* I’ve walked at home instead. I’ve walked 2.5 kilometres in my apartment.* (ID 4, man, age 41–50)

During the lifestyle intervention, being in a safe and understanding environment helped the participants to take part in working towards changing their behaviours. For example, they described that it was helpful to be part of an understanding social climate, meeting others with the same experiences regarding OCD, and being led by healthcare workers that understood mental health problems.

*It’s easy to find excuses to not go. But with* [the lifestyle intervention], *it was easier to go there*,* because of some of these things… people were not… everyone was not super fit and muscular*,* and you knew that everyone there could have a bad day and you could talk about it if you were also having one*,* and that is not possible to do at a regular gym.* (ID 7, man, age 31–40)

Three participants gave examples of increased knowledge facilitating change, such as knowing the results of their blood work or learning what type of exercise fits them best.

*I received a warning when they took a blood test… The test showed high alcohol consumption. That was a wake-up call for me*,* so then I stopped drinking.* (ID 5, woman, age 31–40)

It was also noted that receiving recommendations and knowledge about good healthy behaviours was not always enough, because the barriers to change and engaging in healthy behaviours persisted.

*I’ve tried going to the GP and a dietitian. At the dietitian*,* I felt it was like this: «Don’t eat sugar*,* eat a snack instead» But I’m not stupid. I understand that I shouldn’t eat sugar.* (ID 15, woman, age 31–40)

### OCD is a roadblock in general medical care

Almost all participants (*n* = 15) had comments on how OCD and other mental health issues had impacted their experiences of seeking somatic healthcare.

#### Service-related factors

Most participants (*n* = 13) reflected on service-related factors that had impacted their experiences of seeking general medical care. Some factors were more general, such as the distance to their healthcare centre or differences depending on the healthcare provider.

*Healthcare is not always easy. I mean… it depends on what doctor you have.* (ID 2, woman, age 41–50)

Other factors were more specific to having mental health problems, such as participants reporting not being believed, taken seriously or that their problems were attributed to their mental health diagnosis, even though they had legitimate concerns.

*They don’t believe me. I can’t express… I don’t know what it is. Very often*,* something goes wrong. They don’t believe me*,* but I have real problems. I find that troublesome and sad.* (ID 1, woman, age 51–60)

*I’ve had problems with my stomach for a very long time. The first thing they did was to open my medical record without asking me*,* and I knew that it was locked at that time*,* but whatever. And the first thing they said was: «Are you sure this is not related to your mental problems?»* […] *And it was an ulcer. It… I probably had an ulcer last year too. It was bleeding and I vomited a lot. I was in a lot of pain. I was really suffering.* (ID 13, woman, age 20–30)

There were also examples of positive experiences of service-related factors, such as healthcare personnel adapting to the individuals’ needs.

*They* [hospital staff] *were really amazing at the ward. The most amazing people I’ve met. But it was a bit hard for them too. Because when you are discharged*,* they just want you to go home so they can clean the room for the next person. But then I had to shower and so on. And they were so kind. They arranged that for me. And I told them: « It must be so difficult for you to have me as a patient» But they were like: «No*,* but it’s good that you’re transparent*,* so we know if we’re doing things right. »* (ID 15, woman, age 31–40)

#### Worry and self-doubt

A majority (*n* = 10) of the participants described how worry and self-doubt regarding receiving healthcare affected their experience. They described worrying about not being taken seriously, not being believed or the possibility of being misunderstood or misinterpreted because of their status as a person with OCD or a mental health patient. This seemed to be related to stigma or previous negative experiences within the healthcare service, as mentioned above.

*And then I know that* [the doctor] *said: «No*,* but we can’t do anything about that* [removing that mole]. *It would just be strange» He tried to dismiss it. And when they say strange*,* then you just slump down and think «so there’s something wrong with me*,* I’m mentally unstable» or something like that.* (ID 12, woman, age 51–60)

Five participants also noted that they doubted themselves about whether they were actually sick, or sick enough, to seek help. This in some cases lead to delayed healthcare.

[Previous experiences] *have led to a habit of always questioning myself*,* and maybe question myself for a bit too long. There was this one time… Last year*,* I was taking ADHD* [attention-deficit/hyperactivity disorder] *medication. And I got heart palpitations and chest pain. It was just like… a problem. I called the medical advice hotline and also talked to my mother about it. And I talked to friends and family and called my psychiatric clinic. And everyone said*,* «go to the hospital right now!» And still*,* I just let these problems be for a week and just thought «I’m exaggerating» or «I’m just being dramatic*,* they will just send me home».* (ID 13, woman, age 20–30)

*I sometimes wonder*,* is this real? Do I really need help? Or is it all in my head?* (ID 14, woman, age 31–40)

Further, two participants also noted that they did not want to disclose that they had OCD when seeking healthcare for fear of being misinterpreted or because a perceived lack of knowledge in healthcare services regarding OCD.

*I just tell them that I have problems with anxiety*,* I don’t go into details about having OCD. And I definitely don’t tell anyone at work. I don’t feel safe to do that. I think that even in healthcare*,* if you don’t meet specialists*,* they don’t have that much knowledge of OCD.* (ID 7, man, age 31–40)

#### Complicated compliance

Half of the participants (*n* = 8) reflected on difficulties in complying with medical advice or care because of their OCD. Obsessions and compulsions got in the way of complying with routine care or treatment plans, for example, when providing routine samples as part of monitoring a chronic illness or following medical advice. The effect of OCD lead to increased anxiety, delays or missed controls.

*My dad*,* and I think my grandmother too*,* died of heart problems. And my aunt. So that’s why I do tests every year to check my blood fats and stuff like that. It can be like… sometimes I don’t do it*,* partly because it’s hard to leave my home*,* and partly because it’s so damn unpleasant to do blood tests. And that’s connected partly to my OCD. So yes*,* it makes it more difficult for me to seek health care.* (ID 4, man, age 41–50)

*It’s horrible. It takes me an entire day to do the* [routine stool sample test]. […] *And then I need to go by public transport because I can’t have the sample in my car… Then I need to throw all my clothes – shoes*,* jacket*,* all of it – into the laundry. It’s so hard*,* it turns into this giant project to do it. And that makes me procrastinate. I’ve missed handing in samples as well because I haven’t… so it absolutely affects me.* (ID 15, woman, age 31–40)

*You get skin problems and stuff like that more easily*,* because of washing* [compulsions], *and then on a lot of [creams you are prescribed] it says you shouldn’t use soap* [while using the creams], *but I do it anyway.* (ID 10, man, age 20–30)

### It is not just OCD

Even though all participants could describe how OCD impacted their lifestyle and their ability to seek healthcare, most (*n* = 14) also reported difficulties in identifying and separating the effect of OCD from other factors. These factors included other mental health and somatic comorbidities and other factors such as problems at work or strained social relationships. OCD was at times indistinguishable from the other factors, or they were intertwined. This theme was deemed an overarching theme since participants returned to this reasoning in discussions regarding all above-mentioned themes and subthemes.

*It’s difficult for me to say if it’s the OCD or my overall well-being*,* but the OCD is part of that too.* […] *For me*,* it’s the whole package*,* and that whole package affects me negatively.* (ID 7, man, age 31–40)

Importantly, it was also noted that the different factors affected each other, where worsening problems within one aspect of life, generally affected the rest, resulting in a downwards spiral.

[Besides the OCD, ] *I have pretty severe depression. So it’s absolutely… And then I have ADHD*,* so it’s hard to focus. But it goes hand in hand with my OCD.* [The OCD] *is often the basis of it*,* I think. My depression gets worse when my OCD gets worse*,* and my OCD gets worse when my depression gets worse. And then add ADHD to that*,* and so on.* (ID 10, man, age 20–30)

## Discussion

We explored how OCD affects lifestyle habits and the ability to modify them, as well as experiences of seeking and receiving general medical care, among 16 individuals with OCD and cardiometabolic risk factors that had taken part in the piloting of a lifestyle intervention [23]. The thematic analysis generated three main themes and one overarching theme.

Our participants described both OCD-specific and general barriers to engage in healthy behaviours. These findings lead to the first theme, “Living with multiple barriers to engage in healthy behaviours.” Given that OCD causes functional impairment in many areas of life, including domains related to physical health and lifestyle [3, 33, 34], the finding that OCD was a barrier to implement lifestyle habits was expected. Interestingly, obsessions and compulsions were not only described as direct barriers to making healthy choices, but also indirectly making activities more time-consuming, stressful, troublesome or expensive. The general barriers to engage in healthy lifestyle habits included time, money, stress or bad weather. These barriers have also been found in individuals with other mental disorders and in the general population [35–37]. OCD seemed to exacerbate some of these general barriers, such as being too tired to exercise or not having enough time to engage in healthy behaviours. Hence, OCD creates unique challenges that need to be addressed when designing interventions for this group.

The participants also described difficulties in implementing and maintaining long-term change regarding their lifestyle choices, leading to the second theme: “Lifestyle change – challenging but possible.” Lack of motivation, difficulties in forming and maintaining new habits, and feeling hindered due to OCD or other mental health problems were some of the issues on the way to change that were described, in line with previous research about making lifestyle changes, both in individuals with OCD and in the general population [21, 38]. However, the participants also pointed out possible facilitators to succeed at making lifestyle changes, including the inclusion of individual adaptations, which are important to consider when designing interventions aimed at facilitating change. Participants noted that it was helpful to be in a safe, knowledgeable, and understanding context, which is consistent with what has been found in previous work in the general population, where social support within the intervention and knowledgeable staff have also been acknowledged as important facilitators [38].

The analysis further indicated that, although increased knowledge is important to facilitate change, it is not enough. Health literacy, defined as understanding, appraising, and applying health information to make appropriate health decisions [39], is often mentioned as an important component to make lifestyle changes. Our participants had difficulties applying the health information, rather than low overall health literacy. It is therefore important to consider not only knowledge but application of skills when trying to facilitate change for these individuals, for example in designing interventions and offering medical advice and health care.

The third main theme was “OCD is a roadblock in general medical care.” To our knowledge, this topic has not been explored before in OCD. Some of the factors affecting the experience of seeking healthcare that our participants described were related to the healthcare services. Previous work has shown that individuals with other mental disorders such as schizophrenia are less likely to receive medical examinations and adequate treatments [40]. It has been suggested that this could partly be due to what is known as diagnostic overshadowing, where clinicians attribute somatic symptoms to the patient’s underlying psychiatric condition [40]. This experience is echoed in our material, with participants describing instances of somatic problems being disregarded or attributed to their mental health disorders. This is highly problematic, since individuals with OCD have shown to be at increased risk of developing a myriad of somatic problems and show an increased risk of mortality [6, 7].

Worry and self-doubt affected receipt of healthcare for some participants. This was related to previous negative experiences of seeking healthcare, an effect of self-stigma or internalized stigma, or both [41]. The participants described that, because of their underlying mental health disorder, they did not trust their own experience of somatic symptoms and were therefore reluctant to seek medical advice. They also reported fear of being discriminated or stigmatized by healthcare personnel, preventing them from seeking medical advice for a somatic complaint. This could lead to delayed help-seeking and withholding important information, which, in turn, could have an effect on them receiving adequate diagnosis and treatment. Further, some participants reported that OCD could complicate compliance with medical advice and care, like taking part in medical examinations or routine testing for existing diseases or not being able to follow medical advice. These results indicate a need for increased knowledge regarding OCD among general medical care personnel, and adaptions and help in complying with medical advice tailored to the individuals’ needs. A better integration of services – including improving interprofessional communication, increasing knowledge about psychiatric disorders in general medical care services, and increasing awareness of the management and prevention of somatic conditions in psychiatric settings – is recommended by current guidelines to improve the overall health of individuals with mental disorders [40].

Finally, almost all participants reflected on the overall difficulties trying to separate the effect of OCD from other problems, such as other mental health, physical or social problems. Not only were they difficult to disentangle from each other, but they were also experienced as reciprocally influencing and reinforcing each other. These reflections were prominent throughout the generated main themes, prompting a fourth overarching theme: “It is not just OCD”. Previous research indicates that risk factors related to physical health such as mental health or social problems not only interact but reinforce each other [40, 42], as also shown in our results.

Overall, our findings indicate that the relation between mental health and somatic health is complex and intertwined, which needs to be considered when caring for this patient group in order to improve their physical health [40]. In practice, this would translate into the implementation of more holistic models of healthcare for individuals with OCD that would take into account not only the severity of the psychiatric symptoms but the person’s functioning and well-being as a whole [43].

Several limitations in this study should be noted. Firstly, the questions in the interview guide were clearly directed at finding negative effects and barriers and might not have been sufficiently open as to allow for information of a more general, or positive, nature. Secondly, interpersonal reflexivity [30] could have been improved further. For example, the interview guide was not pilot-tested before use and the final thematic model was not cross-checked by the participants. This might have led to missing important feedback that could have improved the data collection and analysis. Finally, participants in this study had volunteered to participate in a lifestyle intervention where, to be eligible, they had to have several cardiometabolic risk factors, including unhealthy lifestyle habits. Therefore, it is it is important to consider to what other groups this information might be transferable to [44].

## Conclusions

Individuals with OCD experience that their symptoms significantly impact their ability to engage in and maintain healthy lifestyle habits. OCD can also affect healthcare seeking behaviours and experiences of general medical care, which sometimes leads to delayed or low-quality care. These results indicate the need for tailored support for individuals with OCD, as well as the need for a more holistic healthcare model.

## Supplementary Information

Below is the link to the electronic supplementary material.

Supplementary Material 1

## Data Availability

The data that support the findings of this study are not publicly available. The participants were assured that the data would be anonymized, therefore the original transcripts and audio recordings are not available outside of the research group.

## Abbreviations

COREQ: COnsolidated criteria for REporting Qualitative research
LIFT: Swedish acronym for *Livsstilsintervention för tvångssyndrom* [Lifestyle intervention for OCD]
OCD: Obsessive-compulsive disorder
