# Supplementary material for: Lifestyle habits and use of general medical services among individuals with obsessive-compulsive disorder: a qualitative study
Source: BMC Psychiatry. 2026 Mar 16;26:291. doi: 10.1186/s12888-026-07965-7 (PMC13064035; doi:10.1186/s12888-026-07965-7)
Supplement: Supplementary file 1 — Supplementary Material 1 [file 12888_2026_7965_MOESM1_ESM.docx]

**Supplementary material**

**Interview guide Part 2**

1. What made you take part in the LIFT intervention? What did you hope the intervention would lead to?
2. Have you experienced that your OCD has impacted your lifestyle habits? If yes, how?
   1. Physical activity
   2. Diet
   3. Alcohol
   4. Sleep
   5. Tobacco use
3. Does your OCD keep you from making changes regarding health behaviours that you have wanted to make? In what way? During the intervention or in general?
4. Do any other diagnoses, or your mental health, keep you from making changes regarding health behaviours that you have wanted to make? During the intervention or in general?
5. Did your OCD impact the way you were able to take part in the intervention? Would you have needed any other adaptations with regards to your OCD? Are there other things that impacted the way you were able to take part in the intervention?
6. Have you experienced that your OCD, or mental health in general, has kept you from gaining access to somatic health care? In what way?
7. Do you have anything to add?
